# Antigen B from *Echinococcus granulosus* regulates autophagy-mediated macrophage polarization to alleviate immune thrombocytopenia

**DOI:** 10.1186/s13071-025-07182-3

**Published:** 2025-12-15

**Authors:** Hai-chen Song, Dan-lu Li, Jia-jing Wang, Hong-jie Jiao, Ming-wei Li, Li Zhao, Xue-hua Yang, Mei Yan

**Affiliations:** 1 Department of Internal Pediatrics, First Affiliated Hospital of Xinjiang Medical University, Urumqi, 830054 Xinjiang Uygur Autonomous Region China; 2 The Academy of Pediatrics of Xinjiang Medical University, Urumqi, 830054 Xinjiang Uygur Autonomous Region China

**Keywords:** *Echinococcus granulosus*, Antigen B, Immune thrombocytopenia, Autophagy, Macrophage

## Abstract

**Background:**

Immune thrombocytopenia (ITP) is an acquired autoimmune disease characterized by a low platelet count (< 100 × 10^9^/L) induced by an autoimmune mechanism, which increases platelet clearing by macrophages. Antigen B (AgB) is a lipoprotein derived from *Echinococcus granulosus* larvae and has been observed to modulate host immunity. This study evaluated the mechanistic impact of AgB on macrophage polarization in the ITP mouse model.

**Methods:**

This study analyzed blood samples acquired from pediatric patients with ITP and healthy controls. Furthermore, the levels of inflammatory cytokines in plasma, as well as macrophage surface markers and autophagy-related markers [microtubule-associated protein 1 light chain 3 (LC3) and sequestosome-1 (p62)] in peripheral blood mononuclear cells (PBMCs) were evaluated. Moreover, the ITP model was successfully established after immunization with an anti-CD41 antibody and treatment with AgB in vivo. Platelet counts and hemorrhagic symptoms were continuously examined, while plasma inflammatory cytokine levels and the expression of pertinent indicators in the spleen were assessed. RAW264.7 macrophages and lipopolysaccharide (LPS)-stimulated RAW264.7 macrophages were treated with AgB to assess the expression of relevant markers in an in vitro experiment. The mechanism by which AgB regulates LC3 and p62 levels to inhibit LPS-induced macrophages was investigated. Lastly, autophagy inhibitors were administered to evaluate the specific stage of autophagy affected by AgB.

**Results:**

AgB ameliorated hemorrhage and increased platelet counts in ITP murine models while decreasing the M1/M2 macrophage ratio. AgB therapy increased macrophage autophagic flux in vivo and in vitro. To elucidate the effects of AgB on various stages of autophagy, macrophages were treated with two autophagy inhibitors: 3-methyladenine (3-MA) and bafilomycin A1. This study revealed that AgB primarily acts by influencing the expression of LC3II/LC3I and p62, increasing the formation of autophagosomes and enabling lysosomes to identify and consume autophagosomes more accurately. AgB also inhibits macrophage polarization towards M1. These results suggested that AgB reduced hemorrhage in the ITP mouse model by regulating autophagy-mediated macrophage polarization.

**Conclusions:**

This study showed that AgB alleviates ITP by restoring autophagy flux, inhibiting M1 macrophage polarization, and modulating immunity.

**Graphical Abstract:**

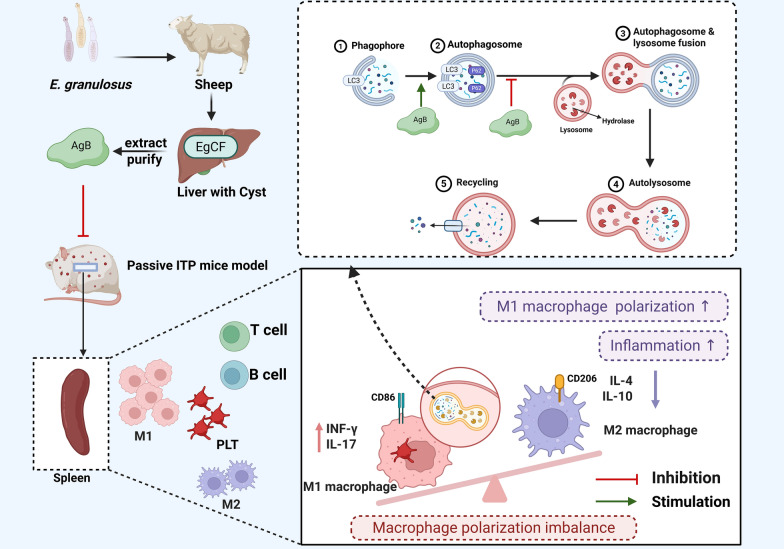

**Supplementary Information:**

The online version contains supplementary material available at 10.1186/s13071-025-07182-3.

## Background

Immune thrombocytopenia (ITP) is a common autoimmune hemorrhagic condition in children, characterized by diminished platelet counts and an increased risk of bleeding [1]. The clinical manifestations of ITP vary from dermatological symptoms (petechiae, purpura, and ecchymosis) to potentially fatal cerebral bleeding, especially when platelet counts fall below 100 × 10^9^/L [2]. ITP pathophysiology involves complex mechanisms, wherein splenic macrophages mediate the phagocytosis of opsonized platelets.

Macrophages can polarize into the classically activated pro-inflammatory M1 type and, alternatively, the activated anti-inflammatory/reparative M2 phenotype [3]. In ITP, M1 macrophages accelerate platelet decrease via phagocytosis and proinflammatory cytokine (tumor necrosis factor (TNF)-α, interleukin (IL)-1β, and IL-6, etc.) release. M2 macrophages, however, protect platelets by releasing IL-10 and transforming growth factor (TGF)-β to mitigate inflammation and facilitate megakaryocyte formation. Thus, ITP progression or remission may be indicated by M1 and M2 macrophage subtypes [4]. Therefore, modulating the M1/M2 balance represents a potential therapeutic approach for ITP.

Autophagy, a lysosomal catabolic pathway that degrades damaged organelles and proteins, maintains adult stem cell self-renewal and differentiation [5]. Several studies have indicated that autophagic dysfunction is associated with ITP pathogenesis. Compared with healthy children, newly diagnosed patients with ITP had a significantly reduced number of Treg cells, as well as autophagy proteins Atg5 and Atg7 [6]. Another study employed an Atg7 knockout mouse and found that autophagy promotes the formation and differentiation of megakaryocytes [7], as well as affecting platelet generation and aggregation [8]. A recent study by Smith et al. [9] also reported dysregulated autophagy in pediatric patients with ITP, demonstrating significant upregulation of the key autophagy markers Beclin-1 and LC3, as well as downregulation of p62, indicating the activation of autophagic flux. Notably, their study observed a marked correlation between this autophagic abnormality and the production of anti-platelet antibodies. Furthermore, autophagy modulates macrophage polarization in autoimmune conditions, such as asthma and rheumatoid arthritis [10, 11]. Thus, autophagy regulation could shift macrophage polarity, which can serve as a therapeutic strategy for ITP.

Cystic echinococcosis (CE) is a zoonotic disease caused by *Echinococcus granulosus* larvae. It represents a substantial disease burden with implications for both global health and economics [12]. Some parasitic protozoa have evolved methods to circumvent the host immune system, thus enhancing their invasion and reproduction [13]. A recent study has identified certain parasites that may protect against allergic/autoimmune disorders, including multiple sclerosis, type 1 diabetes, and inflammatory bowel disease (IBD) [14]. *E. granulosus* infection promotes the development of multiple cysts, predominantly in hepatic and pulmonary tissues [15]. *E. granulosus* cyst fluid (EgCF) comprises proteins that inhibit macrophage inflammatory responses, thus aiding in immune evasion [16].

EgCF modulates C-type lectin receptors CLEC9A and CD205 in the host’s dendritic cells (DCs) and stimulates the catabolic pathway of autophagy. This EgCF-induced autophagy regulates subsequent T-cell responses [17]. Moreover, EgCF substantially promoted LC3 accumulation in autophagosome-like vesicles in DCs [18]. Antigen B (AgB), the primary constituent of proteins secreted in the EgCF [19], is a polymeric lipoprotein (120–160 kDa) with high specificity and immunogenicity, making it valuable for immunodiagnosis [20]. It has been suggested that AgB’s therapeutic potential against IBD involves regulating the intestinal microbiome and M2 macrophage differentiation [21]. Moreover, AgB binds to monocytes/macrophages to suppress their pro-inflammatory activity, indicating an immunomodulatory function [22].

Our previous research demonstrated that AgB inhibits Toll-like receptor 4 (TLR4) endocytosis and CD14 recycling, modulating the activation of the NF-κB and IRF3 pathways to suppress macrophage phagocytosis in the ITP mouse model [23]. However, the comprehensive molecular mechanism by which AgB alleviates ITP remains elusive. Therefore, this study evaluated how AgB affects ITP via autophagy and macrophage polarization using a passive ITP mouse model and lipopolysaccharide (LPS)-induced macrophages.

## Methods

### Children’s peripheral blood

The peripheral blood samples were collected from children with ITP and healthy individuals admitted at the Pediatrics Department and Health Center of the First Affiliated Hospital, Xinjiang Medical University, from 1 February 2022 to 1 February 2024 (Table 1).

**Table 1:** Comparison of basic data of each group

Group	*n*	Male/female	Age (year)	Platelet count (× 10^9^/L)
Control	40	25/15	8.1 ± 1.69	198.56 ± 41.83
ITP	44	24/20	7.3 ± 2.36	47.95 ± 28.14^*^
Newly diagnosed	22	12/10	7.6 ± 2.61	56.42 ± 27.42^*^
Persistent	16	10/6	7.2 ± 1.95	47.13 ± 27.67^*^
Chronic	6	2/4	6.0 ± 1.33	19.17 ± 11.13^*△#^

^*^P < 0.05 compared with the control group

^△^*P* < 0.05 compared with the newly diagnosed group

^#^*P* < 0.05 compared with the persistent group

Children in the ITP group were diagnosed according to the ITP guidelines [2]. ITP stages: newly diagnosed (0–3 months), persistent (3–12 months), and chronic (> 12 months). The blood samples (4 mL/child) were collected into EDTA-anticoagulant tubes, centrifuged at 4 °C at 200 × *g* for 10 min to isolate plasma. The blood cells were then diluted with normal saline (8 mL) and gently layered onto the surface of the lymphocyte separation medium (Solarbio, Beijing, China) in two 15 mL centrifuge tubes. The lymphocytes were then separated via centrifugation at 4 °C at 400 × *g* for 20 min, followed by erythrocyte lysis three times. The remaining peripheral blood mononuclear cells (PBMCs) were frozen at an ultralow temperature at −80℃, followed by −20℃.

**Table 2:** List of primer sequences

Gene	Primer sequence	Species
*CD86*	F: CTGCTCATCTATACACGGTTACC R: GGAAACGTCGTACAGTTCTGTG	Human
*iNOS*	F: TCCAAGGTATCCTGGAGCGA R: CAGGGACGGGAACTCCTCTA	Human
*CD206*	F: GCCTCGTTGTTTTGCGTCTT R: GAGAACAGCACCCGGAATGA	Human
*Arg-1*	F: ACTTAAAGAACAAGAGTGTGATGTG R: GTCCACGTCTCTCAAGCCAA	Human
*LC3*	F: GATGTCCGACTTATTCGAGAGC R: TTGAGCTGTAAGCGCCTTCTA	Human
*p62*	F: GGGGACTTGGTTGCCTTTT R: CAGCCATCGCAG ATCACATT	Human
*GAPDH*	F: AATGGGCAGCCGTTAGGAAA R: GCGCCCAATACGACCAAATC	Human
*CD86*	F: TCAATGGGACTGCATATCTGCC R: GCCAAAATACTACCAGCTCACT	Mouse
*CD206*	F: CTCTGTTCAGCTATTGGACGC R: TGGCACTCCCAAACATAATTTGA	Mouse
*LC3*	F: GAGCGAGTTGGTCAAGATCATCCG R: CATAGATGTCAGCGATGGGTGTGG	Mouse
*p62*	F: AGGAGGAGACGATGACTGGACAC R: AGGCAGTGGCGGCTCCTATTC	Mouse
*GAPDH*	F: AACCCTTAAGAGGGATGCTGC R: TCTACGGGACGAGAAACAC	Mouse

### Passive ITP mouse model

The passive ITP mouse model was established by following the method described previously [24]. For all the analyses, 24 age-matched female BALB/c mice were selected and randomly divided into four groups. (1) Control (Ctrl) group: mice were injected intraperitoneally (i.p.) with 200 μL phosphate-buffered saline (PBS) from day 1 to 5 and then from day 8 to 12. (2) ITP group: received i.p. injections containing 200 μL PBS from days 1 to 5, followed by i.p. injections of 2 μg rat-anti-mouse CD41 monoclonal antibody (mAb) in 200 μL PBS from days 8 to 12. (3) AgB group: received i.p. injections of 100 μg/day AgB in 200 μL PBS from days 1 to 5, followed by i.p. injections of 200 μL PBS from days 8 to 12. (4) AgB + ITP group: received i.p. injection of 100 μg/day AgB in 200 μL PBS from day 1 to 5, followed by i.p. injections of 2 μg anti-CD41 mAb in 200 μL PBS from day 8 to 12. The mice were then euthanized via cervical dislocation after orbital blood collection on day 13, and the liver and spleen were dissected and weighed. The ITP progression analysis included regular assessments of platelet counts and bleeding symptoms.

Ethical approval: all animal protocols were approved by the Animal Research Ethics Committee of the First Affiliated Hospital of Xinjiang Medical University (approval no. 20210301–11). All procedures were performed in accordance with the institution’s guidelines for the care and use of laboratory animals.

Platelet counts were determined using a MindRay BC-5300 Hematology Analyzer (Shenzhen, China). Tail vein blood samples (5 μL) were collected via sterile needle into heparinized tubes and diluted 1:9 in PBS.

The liver and spleen index for each mouse was determined as follows: organ index = liver/spleen weight × 100 (mg)/body weight (g).

### Preparation of AgB

EgCF was obtained from ovine liver echinococcal cysts sourced from the Urumqi slaughterhouse (Xinjiang Uyghur Autonomous Region, China). AgB was purified from EgCF using a previously published method [25].

### Cell culture and treatment

The RAW264.7 macrophage cell line (Peking Union Medical College Cell Resource Center, Beijing) was cultured in Dulbecco’s modified Eagle medium (DMEM) augmented with 10% heat-inactivated fetal bovine serum, 100 U/mL penicillin, and 100 μg/mL streptomycin (Gibco, USA) at 37 ℃ and 5% CO_2_.

An LPS-induced inflammatory model was established to evaluate the effects of AgB on polarization and autophagy of macrophages. The LPS-induced inflammatory model was established by treating RAW264.7 macrophages with 100 ng/mL LPS for 24 h. While this treatment induces an inflammatory response, it does not necessarily lead to complete M1 polarization, which requires additional markers and validation. The RAW264.7-derived macrophages without any induction (M0) were categorized into four groups (2 × 10^6^ cells/well): M0 (M0 macrophages with no treatment for 24 h), M0 + AgB (M0 macrophages with AgB 1000 ng/mL treatment for 24 h), LPS (M0 macrophages with 100 ng/mL LPS treatment for 24 h), and LPS + AgB (100 ng/mL LPS + AgB 1000 ng/mL treatment for 24 h) group. The cell supernatants were collected after the treatment, and their IL-6, TNF-α, and IL-10 levels were evaluated through an enzyme-linked immunosorbent assay (ELISA). Furthermore, cells were collected to assess polarization markers (CD86 and CD206), autophagy flux (LC3II/LC3I ratio and p62), and related proteins.

### Incubation of macrophages with 3-MA

The specific autophagy inhibitor, 3-MA, blocks autophagosome formation. LPS-induced RAW264.7 cells were pretreated with 5 mM 3-MA (Sigma–Aldrich, USA) at 37 ℃ in 5% CO_2_ for 6 h, as per a previous protocol [26]. After PBS washing, cells were cultured in fresh complete DMEM. They were then exposed to either AgB (1000 ng/mL, diluted in PBS) or an equal volume of PBS alone as vehicle control for 24 h, followed by supernatant collection for IL-6 and TNF-α analysis via ELISA. The levels of autophagic flux-related proteins (LC3II/LC3I ratio and p62) in the pelleted cells were assessed using western blotting (WB).

### Incubation of macrophages with bafilomycin A1

Bafilomycin A1 is a vacuolar H^+^-ATPase inhibitor, which prevents lysosomal acidification and autophagosome fusion with lysosomes, blocking LC3II degradation. LPS-induced RAW264.7 cells were pretreated with 200 nM bafilomycin A1 (Sigma–Aldrich, USA) at 37 ℃ in 5% CO_2_ for 3 h, as described previously [27]. After PBS washing, cells were cultured in fresh complete DMEM. They were then exposed to either AgB (1000 ng/mL, diluted in PBS) or an equal volume of PBS alone as vehicle control, with both treatments administered in the same final volume. The cells were maintained under these conditions for 24 h, followed by supernatant collection for IL-6 and TNF-α analysis via ELISA. The levels of autophagic flux-related proteins (LC3II/LC3I ratio and p62) in the pelleted cells were assessed using WB.

Cell Counting Kit-8 (CCK-8) assays were used to identify the most suitable concentration of AgB for assessing cell proliferation. Furthermore, cell viability was assessed via the EdU method.

### The CCK-8 assay

RAW264.7 macrophages (4 × 10^4^/well) were treated with different AgB concentrations (0–2000 ng/mL) for 24 h. Cell viability was assessed via the CCK-8 assay (Beyotime, Shanghai) per the kit’s protocol. Survival rates were calculated at 450 nm absorbance, measured using a fluorometer.

### EdU staining assays

This assay was carried out to detect cell proliferation. Briefly, RAW264.7 cells (5 × 10^4^/well) were placed on 1% Matrigel-coated 8-well slides (Falcon) and treated with 1000 ng/mL AgB for 5 days. On day 5, cells were pulsed with 10 μM EdU at 37 ℃ for 2 h. Cell proliferation was assessed using an EdU kit (Beyotime, Shanghai) per the manufacturer’s guide. The cells were imaged under a Zeiss Observer microscope with consistent exposure settings and processed identically using ImageJ.

### Flow cytometry

PBMCs from children, as well as lymphocytes and macrophages from mouse spleens (1 × 10^6^ cells/well), were harvested and treated with anti-CD16/CD32 antibodies in the dark for 15 min at 4 ℃. The cells were then stained with FITC-conjugated anti-mouse F4/80 and PE-conjugated anti-mouse CD206 (BD Biosciences, San Jose, CA) in the dark for 30 min at 4 °C using the manufacturer’s protocols. A FACSCalibur system (BD Biosciences) was employed with FlowJo analysis software (Tree Star, San Carlos, CA) for flow cytometry.

### Immunofluorescence

RAW264.7 monocytes were cultured on cover-glass-equipped 12-well plates. After various treatments, cells were washed sequentially with PBS, fixed for 10 min using 4% paraformaldehyde, blocked with 3% bovine serum albumin (BSA)/0.3% Triton X-100 for 1 h, and treated overnight at 4 ℃ with primary antibodies. After PBS washing, the cells were probed with fluorescent-conjugated secondary antibody (1:200) for 1 h at room temperature, counterstained with 4′,6-diamidino-2-phenylindole (DAPI) to visualize nuclei, and imaged using a Leica DMi8 inverted fluorescence microscope. The immunofluorescence intensities of LC3 and p62 (Abcam, Cambridge, UK) were quantified as mean gray values using ImageJ, with the Ctrl group mean fluorescence intensity normalized to 1.

### Western blot

Proteins were extracted from spleen tissues and macrophages using RIPA lysis buffer (Thermo, USA) and quantified using a bicinchoninic acid (BCA) assay kit (Thermo, USA). The proteins were then subjected to 10% sodium dodecyl sulfate polyacrylamide gel electrophoresis (SDS-PAGE) separation, transferred to polyvinylidene fluoride (PVDF) membranes, blocked, and incubated overnight at 4 °C with primary antibodies against LC3 (1:2000), p62 (1:5000), CD86 (1:1000), and β-actin (1:5000). Subsequently, the membranes were treated with HRP-conjugated goat anti-rabbit IgG secondary antibody (1:5000) for 1 h at room temperature. Bands were visualized using electrochemiluminescence (ECL) (Biosharp, China) and quantified with ImageJ. Antibodies were acquired from Proteintech Group, Inc. (Wuhan, China).

### Enzyme-linked immunosorbent assay

The levels of inflammatory cytokines (IL-6, TNF-α, interferon (IFN)-γ, IL-17, IL-4, TGF-β, and IL-10) in conditioned media and plasma were quantified via ELISA (Elabscience Biotechnology, Wuhan, China) as directed by the manufacturer.

### Quantitative real-time reverse transcription PCR (qRT-PCR)

Spleen and macrophage RNA were isolated using TRIzol reagent (Ambion, USA) according to the kit’s protocols. The extracted RNA was quantified and then employed for cDNA synthesis using oligo(dT) primers with the TransScript^®^ One-Step gDNA Removal and cDNA Synthesis SuperMix (TRAN, China). qRT-PCR was carried out using the PerfectStart™ Green QPCR SuperMIX (TRAN, China) on a QuantStudio 6 Flex system (ThermoFisher Scientific, USA). The amplification conditions were: 94 ℃ for 30 s, 94 ℃ for 5 s and 60 ℃ for 30 s for 45 cycles, followed by a melting curve analysis. Gene expression was quantified via 2^−ΔΔCt^ normalization. The primers were purchased from Sangon Biotech Co., Ltd (Shanghai, China). The primers employed in this study are detailed in Table 2.

### Histopathological analysis

Spleen tissues from mice in each group were fixed in 4% paraformaldehyde, embedded in paraffin, and sectioned to a thickness of 4 μm. The sections were stained with hematoxylin and eosin (H&E) following standard protocols. The stained sections were examined and imaged using a light microscope (Nikon, Japan) at 10× and 40× magnification.

### Statistical analysis

All values are depicted as mean ± SEM. For multi-group statistical comparisons, one-way analysis of variance (ANOVA) with Tukey’s post hoc test was carried out, while for intergroup comparisons, an unpaired Student’s *t*-test was carried out. All analyses were performed using GraphPad Prism 9.0 (GraphPad Software Inc., La Jolla, CA). The statistical significance was defined as *P* < 0.05.

## Results

### M1/M2 macrophage polarization imbalance and dysregulated autophagy significantly promote ITP progression in pediatric patients

**Fig. 1:**
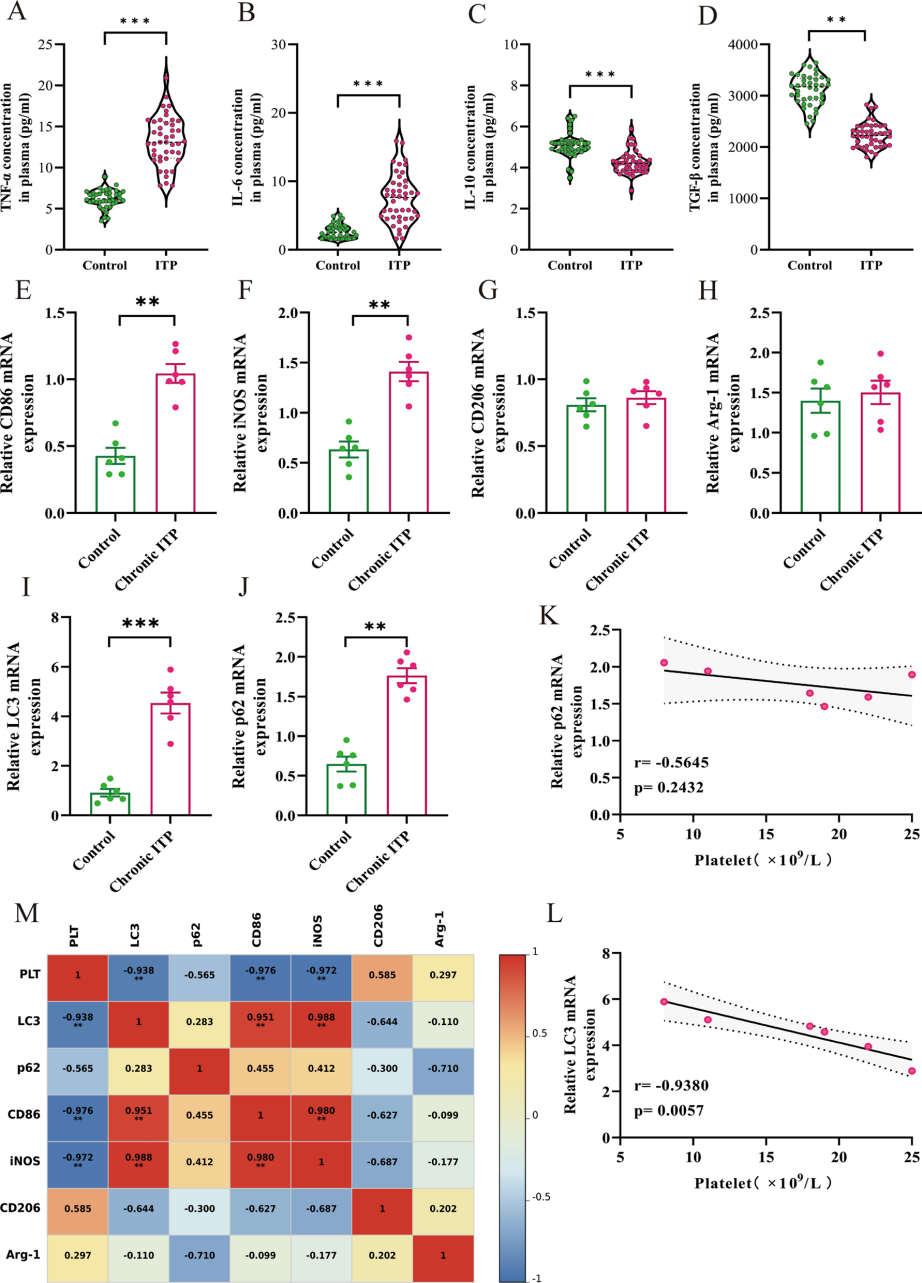
The levels of M1 macrophages and autophagy affect the condition of children with chronic ITP. **A**–**D** The concentrations of M1 macrophage-associated cytokines (TNF-α and IL-6) and M2 macrophage-associated cytokines (IL-10 and TGF-β) in the plasma of the Ctrl (*n* = 40) and ITP (*n* = 44) groups were measured via ELISA. **E**–**J** Quantitative assessment of M1 macrophage surface markers (*CD86* and *iNOS*), M2 macrophage surface markers (*CD206* and *Arg-1*), *LC3*, and *p62* mRNA expression in PBMCs of the Ctrl (*n* = 6) and chronic ITP (*n* = 6) groups. **K**, **L** Correlation between *LC3* and *p62* mRNA expression and platelet count in the chronic ITP group. **M** Associations between platelet count and the mRNA expression of *LC3*, *p62*, *CD86*, *iNOS*, *CD206*, and *Arg-1* in PBMCs of the Ctrl (*n* = 6) and chronic ITP (*n* = 6) groups. Data are expressed as means ± SEM. **P < 0.01, ***P < 0.001

Previous studies have indicated that patients with ITP have increased M1 macrophage activation and impaired M2 polarization relative to healthy controls. Glucocorticoid treatments have been found to alleviate this condition by promoting M2 macrophage polarization [4]. To evaluate plasma levels of cytokines in children with ITP, blood samples were collected from both healthy (*n* = 40) and ITP (*n* = 44) children. The results showed that pro-inflammatory cytokines TNF-α and IL-6 were elevated in the peripheral blood from patients with ITP compared with healthy children (Fig. 1A, B), while anti-inflammatory cytokines IL-10 and TGF-β were significantly reduced (Fig. 1C, D).

To assess whether autophagy and macrophage polarization levels affect ITP progression in children, the mRNA expression of macrophage surface markers and autophagy-related proteins in PBMCs from chronic ITP and healthy children was analyzed. Compared with the healthy children, M1 markers (*CD86* and *iNOS*) were significantly increased in PBMCs from chronic ITP children (Fig. 1E, F), whereas there was no difference in M2 markers (*CD206* and *Arg-1*) (Fig. 1G, H). Furthermore, the transcript levels of autophagy markers (*LC3* and *p62*) were significantly increased relative to Ctrls (Fig. 1I, J). Moreover, there was a negative correlation between platelet count and the relative mRNA levels of *LC3* and *p62* in children with chronic ITP (Fig. 1K, L).

### AgB enhances platelet counts and alleviates thrombocytopenia in ITP mice

**Fig. 2:**
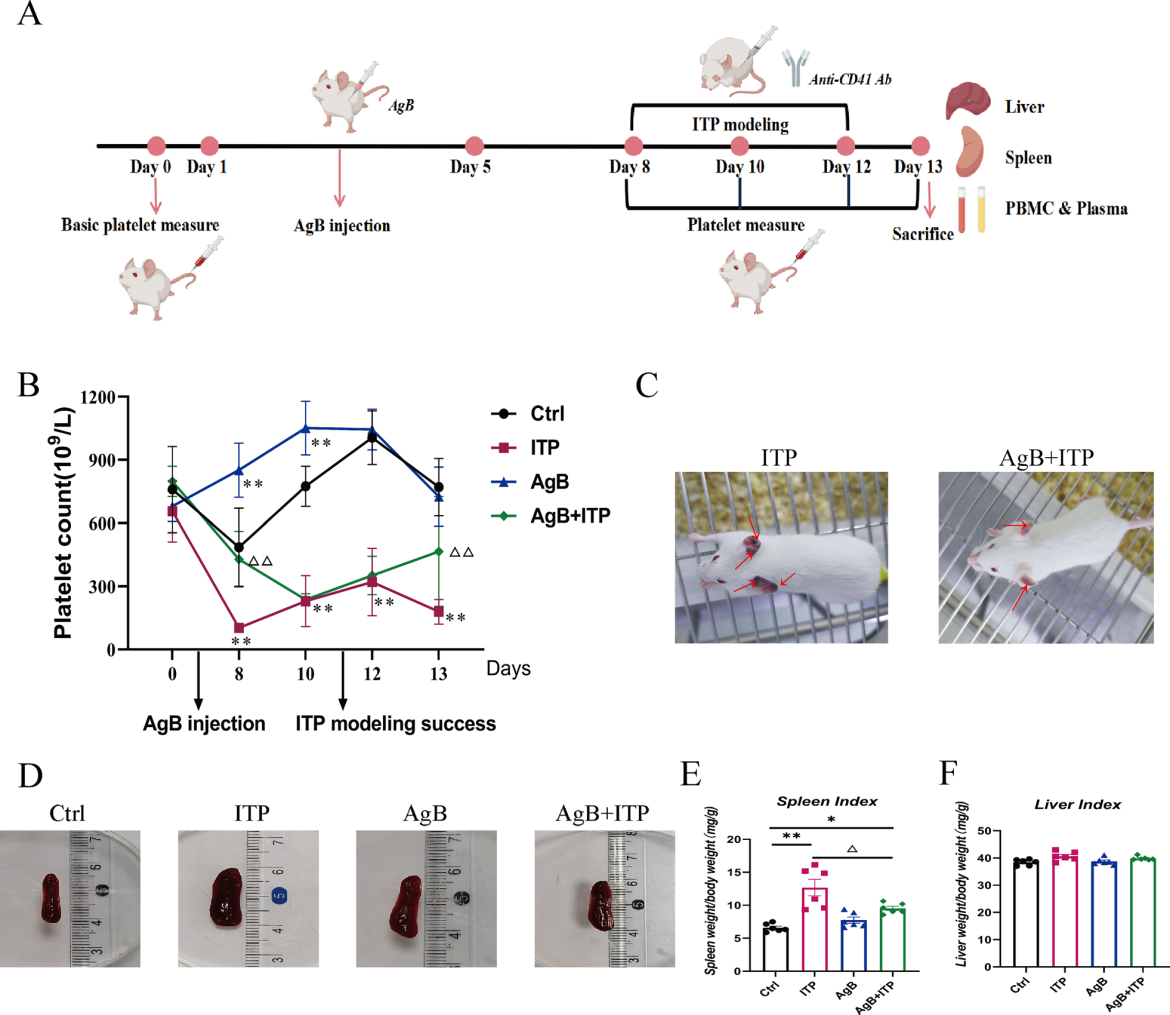
AgB increases platelet counts and reduces hemorrhage in ITP mice. **A** The schematics of murine passive ITP modeling. **B** Platelet count comparison across experimental groups. **C** Mucocutaneous hemorrhage of varying severity in the ITP and AgB + ITP groups. **D** Gross morphological images of the spleen in each group. **E**, **F** Spleen and liver index in each group [organ index = organ weight/body weight × 100 (mg/g)]. Data are expressed as means ± SEM (*n* = 6/group). *P < 0.05, **P < 0.01 (versus Ctrl group). ^△^*P* < 0.05 (versus ITP group)

Following administration of the respective treatments, the peripheral platelet counts of the mice were monitored daily (Fig. 2A). In the passive ITP model, AgB + ITP mice showed an increased platelet recovery compared with the ITP controls during induction (Fig. 2B). In the passive ITP model, the AgB + ITP mice showed an overall trend of platelet recovery during the induction period, with a statistically significant increases in platelet counts compared with the ITP group, particularly on days 8 and 12 (*P* < 0.01) (Fig. 2B). AgB treatment also significantly ameliorated hemorrhage in ITP mouse models (Fig. 2C). In addition, a minor decrease in platelet counts was observed in the Ctrl group, likely attributable to the physiological stress of repeated injections and blood sampling, although this did not affect the overall interpretation of the results.

No mouse died during the entire modeling period. Following i.p. administration of the anti-CD41 antibody in the ITP model group, mice exhibited substantial congestion of the marginal ear vein bilaterally, although no discernible hemorrhagic tendency occurred in other regions. Mice in the AgB + ITP group displayed slight ear vein congestion in both ears; however, the symptoms were less pronounced than those in the ITP group (Fig. 2C).

AgB also affected the morphology, volume, and weight of the spleen in mice. ITP mice exhibited elevated spleen indices but unchanged liver indices compared with the Ctrl group. Spleens showed significant enlargement with severe congestion and marginal infarcts. Compared with the control group, spleens from the ITP mice appeared significantly enlarged with severe congestion and marginal infarcts upon gross examination. When compared with the ITP group, the AgB + ITP group showed a markedly decreased spleen index (*P* < 0.05) although there was no significant difference in the liver index. While spleen volumes in the AgB + ITP group remained slightly increased relative to those in the control group, they were substantially reduced compared with the ITP group (Fig. 2D–F).

Histopathological analysis confirmed the macroscopic observations of splenic congestion. H&E staining revealed that the spleens from the ITP group had marked expansion of red pulp with severe vascular congestion, characterized by dilated splenic sinuses filled with abundant erythrocytes. In contrast, these pathological changes were significantly ameliorated in the AgB + ITP group, with a splenic architecture more closely resembling that of the control group. The AgB group displayed normal splenic histology, indicating no evident toxicity of the treatment itself (Supplementary Fig. S1).

### AgB modulates M1 polarization of macrophages and regulates autophagic flux in ITP mice

**Fig. 3:**
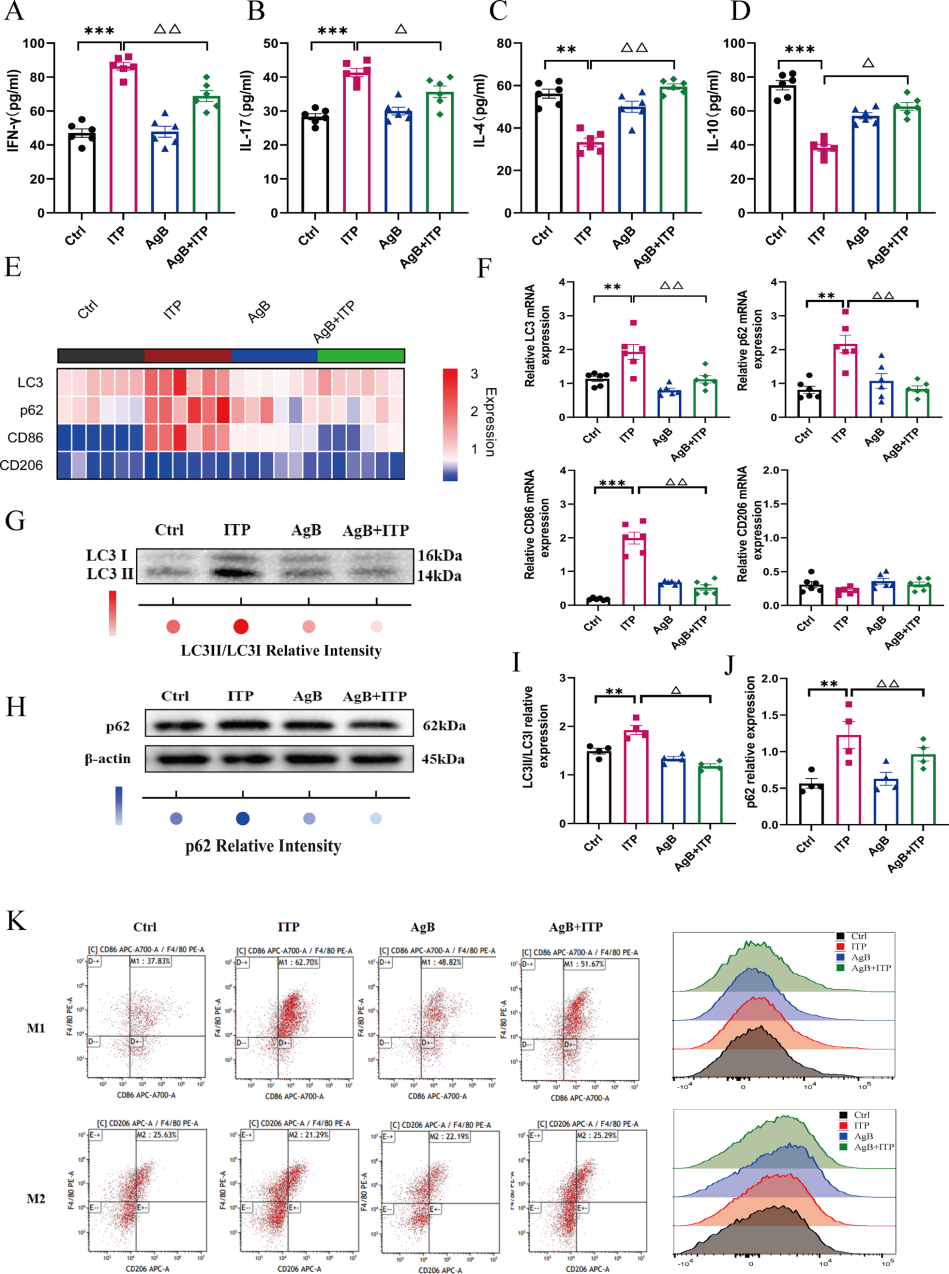
AgB exerts dual immunomodulatory effects by suppressing M1 polarization of macrophages and regulating autophagic flux in ITP mice. **A**–**D** ELISA was performed to evaluate the levels of IFN-γ, IL-17, IL-4, and IL-10 in the plasma of each group. **E** Quantitative assessment of *LC3*, *p62*, *CD86*, and *CD206* mRNA expression levels in spleen tissues of each group (*n* = 6/group). **F** Representative histogram plots from qRT-PCR analysis of *LC3*, *p62*, *CD86*, and *CD206* mRNA expression in the spleen tissues of each group (*n* = 6/group). **G**–**J** The quantitative analysis of LC3II/LC3I and p62 protein levels in the spleen via WB analysis. **K** Representative flow cytometry analysis of M1 macrophages (CD86^+^/F4/80^+^ cells) and M2 macrophages (CD206^+^/F4/80^+^ cells) in spleen tissues of each group (*n* = 6/group). Data are expressed as means ± SEM (*n* = 6/group). **P < 0.01, ***P < 0.001 (versus Ctrl group). ^△^*P* < 0.05. ^△△^*P* < 0.01 (versus ITP group)

On the basis of previous literature on the role of macrophage polarization in ITP pathogenesis, the effects of AgB on this process were evaluated in this study. The results revealed that the cytokines IFN-γ and IL-17 were significantly downregulated in the plasma of the AgB + ITP group (Fig. 3A, B). Furthermore, plasma anti-inflammatory cytokines (IL-4 and IL-10) were also increased in the AgB + ITP group compared with the ITP group (Fig. 3C, D). Moreover, relative to ITP mice, the mRNA expression of an M1 marker (*CD86*), *LC3*, and *p62* were significantly downregulated in AgB + ITP mice; no difference was observed in an M2-associated marker (*CD206*) (Fig. 3E, F). AgB significantly decreased M1 macrophages (CD86^+^/F4/80^+^ cells); however, there was no difference in M2 macrophages (CD206^+^/F4/80^+^ cells) in spleen tissues. These findings demonstrate AgB’s selective suppression of pro-inflammatory M1 pathways (Fig. 3K).

The ITP mice had significantly increased LC3II and p62 protein expression compared with the Ctrl group, suggesting that the autophagy flux was blocked in macrophages from the spleen (Fig. 3E–J). However, AgB intervention significantly downregulated LC3II and p62 levels, indicating that AgB could restore autophagy flux. Moreover, the lymphocytes and macrophages precipitated from the spleen tissues could be mixed. The in vitro verification of whether and at what stage AgB affects the autophagic flux of macrophages requires further assessment.

### AgB inhibits the polarization of macrophages to the M1 type by enhancing macrophage autophagy

**Fig. 4:**
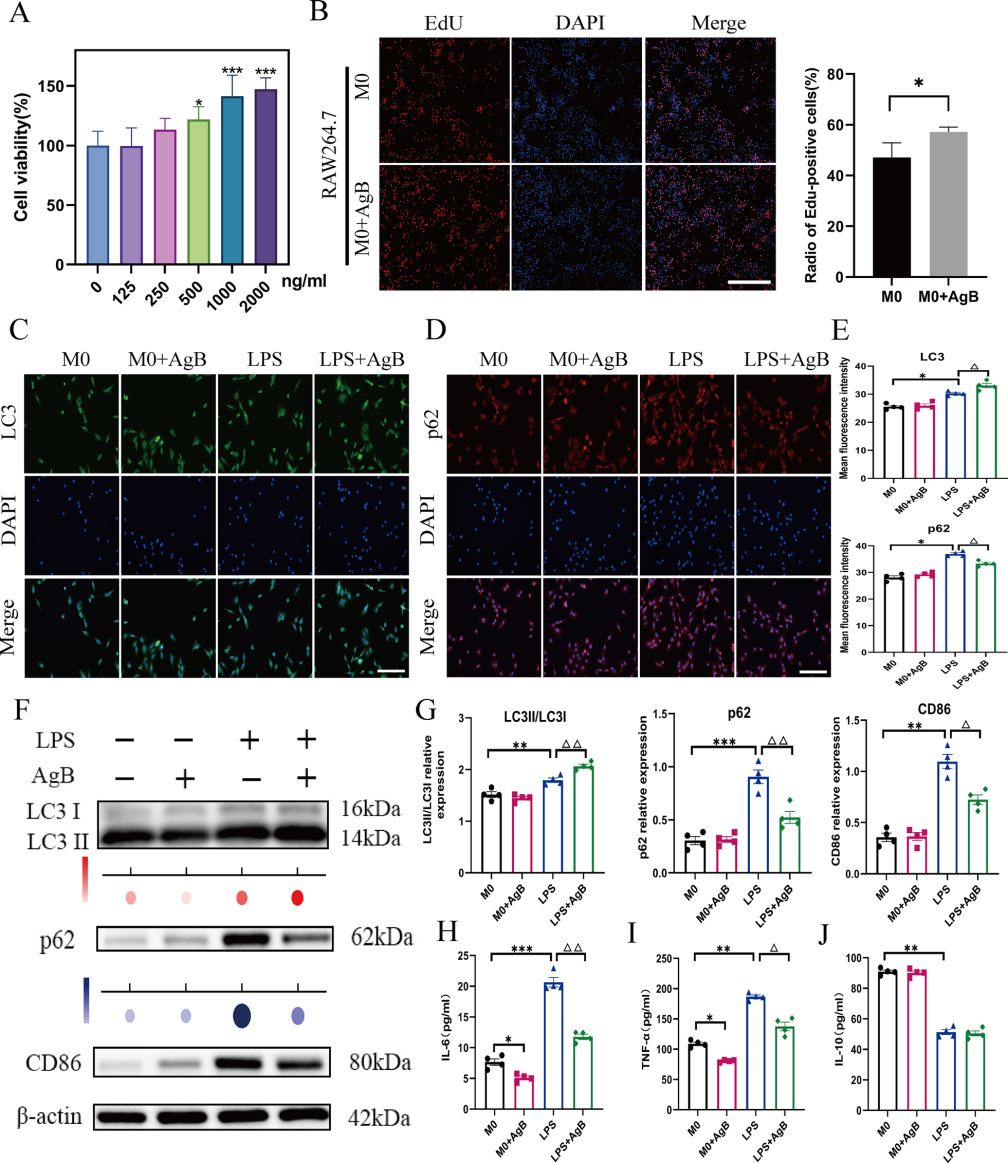
AgB effectively inhibits the release of pro-inflammatory factors by restoring LPS-induced autophagic flux in LPS-induced macrophages. All AgB treatments in panels **B**–**J** were performed using a concentration of 1000 ng/mL. **A** The effect of different AgB concentrations on RAW264.7 cell viability after 24 h. **B** Cell proliferation was tested by EdU assay. Scale bar = 50 µm. **C**–**E** Representative immunofluorescence images and statistical results for the LC3 (green) with p62 (red) in RAW264.7 cells with different treatments (*n* = 4/group). Scale bar = 50 µm. **F**, **G** WB quantification of LC3II/LC3I, p62, and CD86 protein levels. **H**–**J** The IL-6, TNF-α, and IL-10 levels in RAW264.7 cell supernatants were assessed through ELISA. Data are expressed as means ± SEM (*n* = 4/group). *P < 0.05, **P < 0.01, ***P < 0.001 (versus M0 group). ^△^*P* < 0.05. ^△△^*P* < 0.01 (versus M1 group)

To further investigate the effects of AgB in macrophages, RAW264.7 cells were incubated with varying concentrations of AgB (0–2000 ng/mL) for 24 h. The selection of 1000 ng/mL as the optimal AgB concentration was on the basis of the results of the CCK-8 assays (Fig. 4A), which indicated that this concentration led to cell viabilities similar to those after treatment with 2000 ng/mL AgB. Following the principle of using the minimum effective concentration, 1000 ng/mL was chosen for all subsequent experiments. Moreover, an EdU assay was carried out to assess the alterations in cellular physiology, which revealed no significant reduction in cell viability or proliferation in the presence of AgB, indicating the absence of cytotoxic effects (Fig. 4B).

Macrophage autophagy status was assessed via autophagic markers LC3 and p62. LC3II (the lipidated autophagosome marker) is widely used to monitor autophagic activity. The p62 protein serves as a cargo receptor for the autophagic destruction of ubiquitinated substrates, and its accumulation is typically utilized as a marker of impaired autophagy [28]. Immunofluorescence analysis of the autophagy markers LC3 and p62 provided further evidence of the effect of AgB on autophagic flux (Fig. 4C–E). Representative images (Fig. 4C, D) and their corresponding quantification (Fig. 4E) indicated that LPS stimulation significantly increased accumulation of LC3 (green fluorescence) and p62 (red fluorescence). AgB treatment effectively reversed these changes, reducing p62 accumulation and promoting the formation of LC3 puncta, indicative of restored autophagic flux. Here, LPS significantly increased LC3II, p62, and CD86 protein levels relative to the Ctrl, indicating that LPS blocked autophagic flux in LPS-induced macrophages and partially promoted macrophage activation toward a pro-inflammatory phenotype (Fig. 4F, G). However, AgB intervention significantly upregulated LC3II and downregulated p62 levels, promoting autophagosome formation and lysosomal degradation in macrophages, suggesting that AgB could restore LPS-impaired autophagy flux in LPS-induced macrophages. Immunofluorescence analysis showed that AgB significantly enhanced LC3II and reduced p62 in LPS-induced macrophages, further supporting that AgB could activate autophagy in an LPS-induced macrophage model. Moreover, AgB also suppressed M1-associated cytokines (IL-6 and TNF-α) without altering the M2-associated cytokine IL-10 versus the M1 group (Fig. 4H–J).

**Fig. 5:**
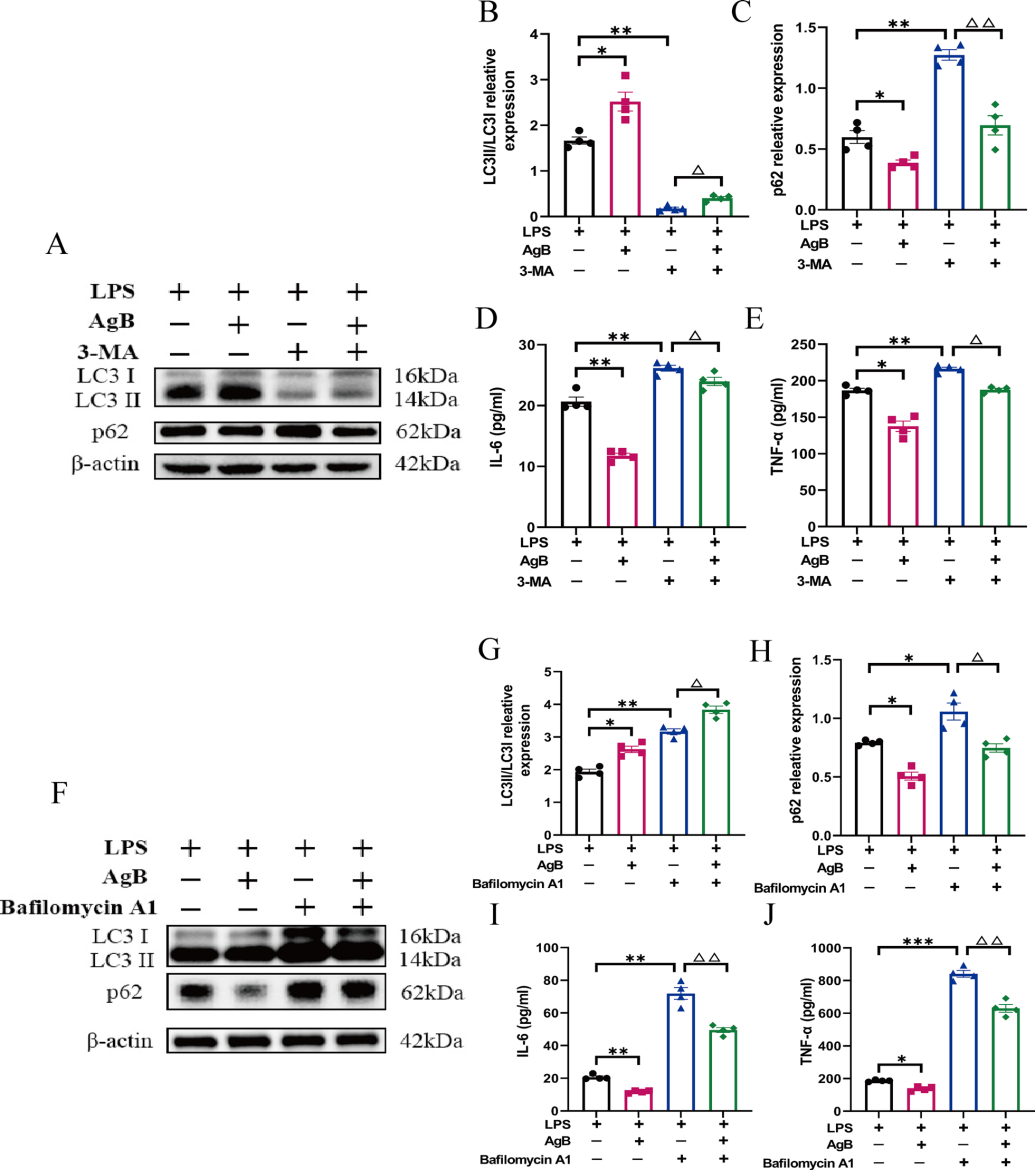
AgB effectively restores the autophagic flux of LPS-induced macrophages through a dual autophagy regulatory mechanism and significantly inhibits the release of pro-inflammatory factors. **A**–**C** WB quantification of LC3II/LC3I and p62 protein levels in 3-MA-treated and untreated Ctrl groups. **D**, **E** IL-6 and TNF-α levels in the supernatant of the 3-MA-treated and untreated Ctrl groups were analyzed via ELISA. **F**–**H** WB quantification of LC3II/LC3I and p62 protein levels in bafilomycin-A1-treated and untreated Ctrl groups. **I**, **J** IL-6 and TNF-α levels in the supernatant of the bafilomycin-A1-treated and untreated Ctrl groups were identified via ELISA. Data are expressed as means ± SEM (*n* = 4/group). *P < 0.05, **P < 0.01, ***P < 0.01 (versus M1 group). ^△^*P* < 0.05. ^△△^*P* < 0.01 (versus corresponding cells treated without 3-MA or bafilomycin A1)

The process of autophagy encompasses four principal stages: (1) nucleation and elongation of isolation membranes (phagophores), (2) autophagosome formation, (3) fusion of autophagosomes with lysosomes to produce autolysosomes, and (4) destruction of terminal cargo [5]. To assess the potential role of AgB-regulated autophagy in macrophage polarization, two autophagy inhibitors targeting distinct pathway stages, 3-MA and bafilomycin A1, were employed [25]. The data revealed that 3-MA pretreatment inhibited LC3II production and prevented p62 degradation compared with the untreated group, demonstrating effective inhibition of LPS-regulated autophagy in M1 macrophages. Moreover, AgB treatment restored LPS-impaired autophagic flux in macrophages, which is evident from the increased LC3II/LC3I ratio and p62 downregulation (Fig. 5A–C). This correlated with reduced M1-associated cytokines (IL-6 and TNF-α) versus LPS + 3-MA groups (Fig. 5D, E). Moreover, the addition of bafilomycin A1, a late-stage autophagy inhibitor that blocks autophagosome–lysosome fusion, facilitated the accumulation of LC3II and p62 proteins relative to the untreated group (Fig. 5F–H). Bafilomycin A1 treatment led to significantly elevated LC3II accumulation in LPS-stimulated macrophages, with a more pronounced effect in AgB-treated cells. Notably, AgB treatment attenuated the bafilomycin-A1-induced p62 accumulation, suggesting that AgB enhanced autophagic flux before the late-stage inhibition. Furthermore, the data indicated that the pro-inflammatory effect of bafilomycin A1 was more pronounced than that of 3-MA, as indicated by higher levels of IL-6 and TNF-α (approximately 65 and 800 pg/mL, respectively; Fig. 5I, J). In addition, AgB treatment significantly lowered the production of these M1-related pro-inflammatory cytokines in LPS-stimulated macrophages even when autophagy was inhibited at different stages of the autophagy pathway by either 3-MA (early stage) or bafilomycin A1 (late stage). It is worth noting that AgB nevertheless reduced IL-6 and TNF-α secretion to some extent even when autophagy was pharmacologically inhibited by either 3-MA or bafilomycin A1 (Fig. 5). This observation indicates that while the restoration of autophagy is an important component of the anti-inflammatory action of AgB, it is not the sole mechanism involved.

## Discussion

This study revealed that M1/M2 macrophage polarization imbalance and dysregulated autophagy are features of ITP in pediatric patients. Furthermore, the role and impact of AgB on the function of autophagy-mediated macrophages in an ITP model were delineated. AgB treatment significantly elevated the platelet count in passive ITP mouse models while inhibiting M1 polarization of macrophages, reducing the secretion of pro-inflammatory factors, alleviating hemorrhagic manifestations, and modulating cellular autophagy levels in vivo. This finding supports the hypothesis that helminth infections may alleviate autoimmune pathology. AgB upregulated LC3II and downregulated p62 in vitro, promoting autophagosome formation and their lysosomal degradation in macrophages, suggesting that AgB could restore LPS-impaired autophagy flux in M1 macrophages.

ITP is an autoimmune bleeding disorder characterized by pediatric thrombocytopenia caused by spleen tyrosine kinase (Syk)-mediated macrophage phagocytosis of antibody-coated platelets [2]. A previous study has indicated that splenic M1 macrophages are elevated in patients with ITP [29]. Recent studies have found that the application of eltrombopag (ELT) [30], high-dose dexamethasone (HD-DXM), all-trans-retinoic acid (ATRA), and low-dose decitabine (DAC) could promote M2 polarization [4, 31], restoring the normal M1/M2 ratio, providing new insights for the treatment of ITP. M1 macrophages express CD86 and iNOS protein and pro-inflammatory cytokines (IL-6, IFN-γ, TNF-α, and IL-17) secretion, whereas M2 phenotypes express CD206 and Arg-1 expression and anti-inflammatory cytokines (IL-10, TGF-β, and IL-4). Peripheral blood samples were collected from 44 ITP and 40 healthy children. ELISA assays revealed significantly elevated levels of M1 macrophage-associated cytokines (TNF-α and IL-6) and decreased M2 macrophage-associated cytokines (IL-10 and TGF-β) levels in patients with ITP compared with Ctrls (Fig. 1A–D). Moreover, for in vitro analysis, a passive ITP mouse model was established. The data were consistent with clinical findings, indicating a significant increase in M1 macrophage ratio in the spleens, elevated M1-related cytokines (IFN-γ and IL-17), and reduced M2-related cytokines (IL-4 and IL-10) in ITP mice (Fig. 3A–D). These findings indicate disrupted M1/M2 macrophage balance in ITP pathogenesis.

AgB is a phosphatidylcholine-rich lipoprotein, classified within the hydrophobic ligand-binding protein family of lysosomal proteins, and plays a crucial role in host–parasite interactions during infection [32]. AgB functions as a possible receptor for binding tissue-resident macrophages, recruiting inflammatory monocytes, and facilitating the polarization of anti-inflammatory macrophages [33]. AgB can also be internalized into macrophages via endocytosis mediated by caveolae/rafts. Furthermore, a minor fraction may enter cells through clathrin-dependent pathways, participating in immunomodulation and lipid transport. Following endocytosis, AgB may localize to distinct cellular compartments and disrupt immune-related activities, including phagocytosis, antigen presentation, cytokine release, and vesicular trafficking [34]. Ultimately, it reaches lysosomes for degradation or recycling, with some cargo being transported back to the plasma membrane.

Several studies have indicated that parasitic infections may offer therapeutic potential for autoimmune disorders [35]. In IBD mouse models, *E. granulosus* infection and AgB decreased peritoneal M1 macrophages (F4/80^+^ and CD11c^+^) and augmented intestinal M2 macrophages (F4/80^+^ and CD206^+^) in the lamina propria [20]. Moreover, AgB effectively inhibits the formation of the Toll-like receptor 4 and myeloid differentiation protein-2 (TLR4–MD2) complex in LPS-induced macrophages, reduces TLR4/TLR4 dimerization, and significantly inhibits TLR4 internalization and CD14 recycling. This results in the modulation of TLR4 signaling, which reduces M1 polarization and macrophage-mediated phagocytosis [23]. However, the specific mechanisms by which AgB acts remain incompletely determined.

Autophagy is a crucial regulatory mechanism in macrophages, ensuring the effective elimination of invading germs and pathogens from living organisms. Macrophages can internalize cells by endocytosis, creating autophagosome structures, which subsequently merge with acidic lysosomes to break down and redistribute pertinent chemicals inside the cell [36]. LC3 primarily consists of two isoforms: LC3I and LC3II. LC3I is predominantly localized in the cytoplasm, whereas LC3II is mainly localized on autophagosomal and autophagy-lysosomal membranes [37]. LC3II functions as a specific marker for autophagosomes and indicates autophagy initiation [38]. During autophagy, soluble LC3I undergoes ubiquitin-like processing and conjugates with phosphatidyl-ethanolamine to form LC3II on autophagy-lysosomal membranes, facilitating autophagosome formation. p62 is a ubiquitin-binding scaffold protein that serves as a regulatory element in autophagosome formation and is destroyed during the intermediate to late phases of autophagy. An elevation in LC3II levels indicates autophagy initiation and autophagosome accumulation. If this is followed by a decrease in p62, it shows unimpaired autophagic flow. Whereas increased levels of both LC3II and p62 imply normal autophagy initiation but impaired downstream autolysosome formation, which may contribute to macrophage polarization toward the M1 phenotype [39]. This study found that an imbalance in macrophage polarization disrupts immune homeostasis, leading to an increased inflammatory response that exacerbates platelet destruction and immune dysregulation in pediatric patients. Moreover, the present study revealed a negative correlation between platelet count and the mRNA expression levels of *LC3* and *p62* in PBMCs from patients with chronic ITP, suggesting that these may serve as indicators of ITP severity as well as bleeding scores for determining hemorrhagic risk.

Overall, the findings indicate that AgB modulates autophagy to suppress M1 macrophage polarization. While previous research has shown that AgB can inhibit TLR4 signaling to reduce M1 polarization and phagocytosis [23], the autophagy pathway identified in this study represents a distinct mechanism. Furthermore, the autophagic flux was blocked in ITP model mice and LPS-induced macrophages; however, AgB treatment effectively restored autophagy flux both in vivo and in vitro. To determine whether this restoration reflects actual autophagy activation or prevention of autophagosome accumulation from flux blockade, the effect of 3-MA and bafilomycin A1 was compared. The western blot analysis of the expression of autophagy indicators revealed that the elevated LC3II/LC3I ratio in LPS-induced macrophages was suppressed by 3-MA, but further augmented by bafilomycin A1. AgB increased the LC3II/LC3I ratio, indicating that AgB promotes autophagy flux (Fig. 5B, G). Furthermore, even with the presence of autophagy inhibitors (3-MA/bafilomycin A1), AgB retained its capacity to downregulate p62 expression in LPS-induced macrophages (Fig. 5C, H). On the basis of previous studies, it was hypothesized that macrophages internalize AgB, which is subsequently degraded by acidic organelles of the endolysosomal system, specifically, the late endosomes, lysosomes, and the product of their fusion, the endolysosomes. This process may enhance lysosomal function or facilitate autophagosome–lysosome fusion, thereby restoring autophagic flux and contributing to the observed downregulation of p62. This indicates how AgB effectively restores the autophagic flux of LPS-induced M1 macrophages via a dual autophagy regulatory mechanism (promoting the early inhibition of 3-MA and collaborating with bafilomycin A1 for late degradation). Furthermore, bafilomycin-A1-treated LPS-induced macrophages had a significantly increased secretion of pro-inflammatory cytokines (IL-6 and TNF-α) in the cell culture supernatant compared with the untreated group (Fig. 5I, J). This bafilomycin-A1-mediated amplification of inflammation may result from lysosomal-rupture-stimulated cellular stress rather than mere autophagy inhibition.

A limitation of this study was the lack of a standardized system for the assessment of bleeding in the ITP mouse models. Although qualitative measures of hemorrhagic symptoms were used, the absence of objective metrics may have affected the precision of evaluating the therapeutic impact of AgB on hemorrhage alleviation. A second limitation is the relatively small size of the mouse sample. On the basis of the findings of this study, future investigations should focus on the establishment of standardized scoring criteria to enable more robust comparisons across studies.

## Conclusions

This study showed that AgB administration increased platelet counts and alleviated hemorrhage in ITP mice. Furthermore, this study confirmed that AgB could restore autophagy flux by inhibiting autophagy activation and accumulation by suppressing autophagic degradation in ITP mice. AgB promotes immunomodulatory effects by suppressing pro-inflammatory cytokine secretion and inhibiting macrophage polarization toward the M1 phenotype. Therefore, AgB could serve as a promising therapeutic candidate for ITP and related autoimmune conditions.

## Supplementary Information

Supplementary material 1. Fig. S1: H&E-stained micrographs of spleen sections in each group at both 10X and 40X magnification. Table S1: Platelet count comparison across experimental groups.

## Data Availability

The data generated or analyzed during this study are included in this published article.
