# Supplementary material for: Antigen B from Echinococcus granulosus regulates autophagy-mediated macrophage polarization to alleviate immune thrombocytopenia
Source: Parasit Vectors. 2025 Dec 15;19:38. doi: 10.1186/s13071-025-07182-3 (PMC12822193; doi:10.1186/s13071-025-07182-3)
Supplement: Supplementary file 1 — Supplementary material 1. Fig. S1: H&E-stained micrographs of spleen sections in each group at both 10X and 40X magnification. Table S1: Platelet count comparison across experimental groups. [file 13071_2025_7182_MOESM1_ESM.doc]

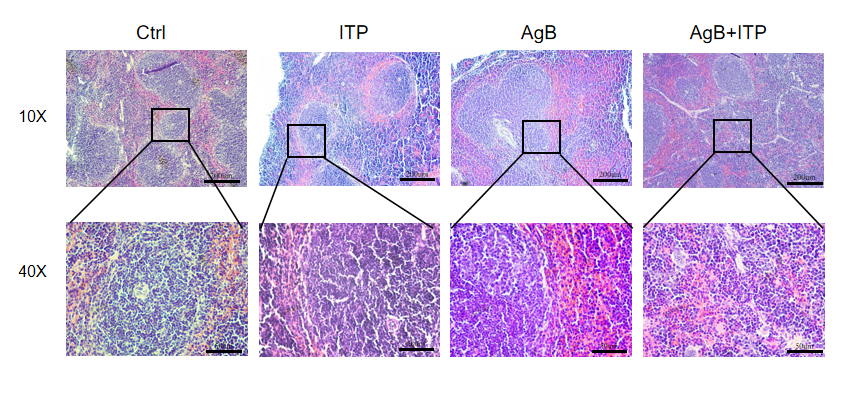


**Fig. S1:** H&E-stained micrographs of spleen sections in each group at both 10X and 40X magnification. Scale bar = 200/50 µm.

**Table S1** Platelet counts of each group of mice

|  | d0 | d8 | d10 | d12 | d13 |
| --- | --- | --- | --- | --- | --- |
| Ctrl | 759.1±164.2 | 485.4±185.2 | 774.4±94.7 | 1005.0±44.8 | 758.9±184.2 |
| ITP | 655.6±145.5 | 102.1±17.9** | 229.7±121.1** | 320.6±26.6** | 179.1±58.7** |
| nAgB | 679.7±72.1 | 851.0±128.5** | 1051±127.6** | 1044.1±33.8 | 725.4±139.8 |
| AgB+ITP | 797.6±71.5 | 428.6±131.6^△△^ | 236.1±27.8 | 351.4±31.8 | 465.4±160.3^△△^ |

**Table S1** Platelet count comparison across experimental groups. Data were expressed as mean ± SEM (n = 6/group). ***p < 0.01* (versus Ctrl group); ^△△^*p < 0.05* (versus ITP group).
